# Upregulation of ADAM12 Is Associated With a Poor Survival and Immune Cell Infiltration in Colon Adenocarcinoma

**DOI:** 10.3389/fonc.2021.729230

**Published:** 2021-09-16

**Authors:** Zigao Huang, Hao Lai, Jiankun Liao, Jinghua Cai, Baojia Li, Linghou Meng, Wentao Wang, Xianwei Mo, Haiquan Qin

**Affiliations:** ^1^Guangxi Clinical Research Center for Colorectal Cancer, Guangxi Cancer Hospital and Guangxi Medical University Affiliated Cancer Hospital, Nanning, China; ^2^Division of Colorectal & Anal Surgery, Department of Gastrointestinal Surgery, Guangxi Medical University Cancer Hospital, Nanning, China

**Keywords:** ADAM12, colorectal adenocarcinoma, prognosis, immune infiltration, biomarker

## Abstract

**Background:**

A disintegrin and metalloprotease 12 (ADAM12) is a member of the multidomain protein family, but the mechanisms by which it affects prognosis and immune cell infiltration in patients with colon adenocarcinoma (COAD) remain unclear. Here, our study aimed to analyze the prognostic value of ADAM12 and investigate the correlation between ADAM12 expression and immune cell infiltration in patients with COAD.

**Methods:**

Differential expression analyses were performed using the Oncomine and UALCAN databases, and prognostic analyses were conducted using PrognoScan, Gene Expression Profiling Interactive Analysis (GEPIA), and Kaplan–Meier Plotter. Then, the cBioPortal database was used to analyze alterations in the ADAM12 gene, and the STRING and Metascape websites were used to conduct Gene Ontology and Kyoto Encyclopedia of Genes and Genomes analyses. Additionally, relationships between ADAM12 and the immune microenvironment were evaluated based on the TIMER, GEPIA, and TISIDB databases.

**Results:**

ADAM12 was overexpressed in COAD tissues, and higher ADAM12 expression correlated with a worse prognosis for patients with COAD. The gene regulatory network suggested that ADAM12 was mainly enriched in extracellular matrix (ECM) organization, ECM proteoglycans, skeletal system development, and ossification, among other pathways. Moreover, ADAM12 expression significantly correlated with the abundance of CD4+ T cells, B cells, CD8+ T cells, neutrophils, macrophages, dendritic cells, and their markers, as well as lymphocytes, immunomodulators, and chemokines.

**Conclusions:**

In colorectal tumors, ADAM12 may play vital roles in regulating the ECM and the recruitment of immune cells, and we suggest that ADAM12 will become a reliable biomarker for determining response to immunotherapy and the prognosis of patients with COAD.

## Introduction

According to the latest global cancer survey, colorectal cancer (CRC), a highly heterogeneous disease, is the third most common malignant tumor worldwide (1). The morbidity and mortality rates of CRC are increasing globally annually, especially in developing countries such as China, Brazil, and India, where CRC-related morbidity and mortality rates are increasing 20% each year (2). This disease poses a major challenge to global health. Furthermore, despite significant advances in cancer-related treatment techniques, overall survival (OS) rates for patients with CRC remain unsatisfactory. The vast majority of patients are diagnosed with CRC at an advanced stage, and the tumor often simultaneously invades or metastasizes to other organs. Therefore, an analysis of the pathogenesis of CRC and the identification of potentially reliable biomarkers for prognosis and treatment are urgently needed.

The “a disintegrin and metalloproteases” (ADAMs) are a metalloproteinase family of transmembrane glycoproteins containing metalloproteinase and disintegrin domains. To date, approximately 25 members have been discovered. ADAM12 is an important member of the ADAM family (3). Previous studies have identified a vital role for ADAMs in cancer development, and these proteases regulate the secretion of insulin-like growth factors, transforming growth factor α, and epidermal growth factor (4–7). As a member of the ADAM family, ADAM12, like other members, is expressed at high levels in tumor tissues, and lower expression is associated with a better survival prognosis (8, 9). Wang et al. (10) first reported that ADAM12 expression in human pituitary adenomas is associated with the epithelial-mesenchymal transition (EMT). Interestingly, subsequent studies also indicated that ADAM12 induced EMT in pituitary adenomas through the EGFR/ERK signaling pathway and promoted cell migration, invasion, and proliferation. Additionally, Park and colleagues found that ADAM12 expression is closely related to the occurrence and progression of CRC, and ADAM12 knockdown induced strong antitumor activity in mouse models (11). In addition, the expression profile of ADAM12 and its potential prognostic value and therapeutic targets in different cancers, including thyroid cancer (12), lung adenocarcinoma (13), and pancreatic adenocarcinoma (14), have been reported. However, few studies have examined ADAM12 expression in colorectal adenocarcinoma (COAD), its prognostic value, or its relationship with immune cell infiltration.

In the present study, we comprehensively analyzed the abnormal expression and prognostic value of ADAM12 in patients with COAD using multiple publicly available databases, including Oncomine, UALCAN, PrognoScan, GEPIA, and Kaplan–Meier Plotter. Signaling pathways involved in the effects of ADAM12 were investigated using the STRING and Metascape interactive online websites to perform a functional enrichment analysis and investigate the potential molecular mechanism of the ADAM12 gene in tumor progression. Additionally, the correlation of the immune microenvironment was explored using the TIMER, GEPIA, and TISIDB databases. Our research focused on identifying ADAM12 as an immune-related marker that is potentially useful to guide immunotherapy in patients with COAD.

## Materials and Methods

### Bioinformatic Analysis of ADAM12 Gene Expression

Oncomine (www.oncomine.org), a practical online platform containing 715 datasets and 86,733 samples for expression analyses of target genes (15, 16), was used to compare the expression of ADAM12 mRNA between CRC and matched normal tissues. First, we entered the ADAM12 gene into the search box and obtained the expression profiles of ADAM12 across cancers. Second, “cancer *vs*. normal analysis” was selected, and the “cancer type” was limited to CRC. Meanwhile, the threshold was set to p=0.05 and fold change=1.5 by default. Finally, statistical significance was obtained directly from the corresponding web analysis outcomes.

UALCAN (http://ualcan.path.uab.edu/index.html), a comprehensive and user-friendly website for the analysis of cancer data (17), was used to examine ADAM12 expression in human cancers and to evaluate the promoter methylation level in COAD samples in TCGA. Additionally, we also analyzed the effects of different clinical characteristics on ADAM12 expression, such as age, sex, weight, stage, race, TP53 mutation status, histological subtype, and nodal metastasis status. Moreover, we supplemented the effect of the status of four DNA mismatch repair proteins (MLH1, PMS2, MSH2, and MSH6) on ADAM12 expression *via* TIMER2.0 (http://timer.cistrome.org/) (18, 19).

Immunohistochemical staining for the ADAM12 protein in colorectal tissue was obtained from the Human Protein Atlas (https://www.proteinatlas.org/). In addition, its protein model and 3D structure were explored using the SWISS MODEL (https://swissmodel.expasy.org/) (20).

### Prognostic Survival Analysis

An analysis of the association of specific genes with the survival of patients with COAD was conducted using Gene Expression Profiling Interactive Analysis (GEPIA), PrognoScan, and Kaplan–Meier Plotter. GEPIA (http://gepia.cancer-pku.cn/index.html), a comprehensive and interactive web server (21, 22), provided us with associations between ADAM12 expression and prognosis. PrognoScan (http://www.prognoscan.org/), a new database for meta-analyses of the relation between gene expression and prognosis (23), was used for the survival analysis in our study. The Kaplan–Meier Plotter database (https://kmplot.com/analysis/), an open website designed to evaluate the effect of genes on the survival prognosis of patients with different cancers (24), was applied to further validate our survival analysis results. Patients with COAD were divided into two groups based on the median expression of the specific gene (high vs. low expression) for further prognostic analysis. Hazard ratios (HRs; 95% confidence intervals) and P values were determined for survival, and P < 0.05 served as the significance threshold.

### cBioPortal Database

The cBioPortal database (www.cbioportal.org), an open web resource for analysis (25), was used to analyze alterations in the ADAM12 gene. We chose the “Query” module, selected “DFCI, Cell Reports 2016,” “TCGA, Firehose Legacy,” and “TCGA PanCancer Atlas” in the “bowel” section, clicked the “Query by gene” button, and entered the “ADAM12” gene. The results of structural variant data, mutation data, and CNA data are shown in the “Cancer Types Summary” module. The results of the analysis of ADAM12 mutations are shown in a schematic diagram, and its 3D structure was obtained by clicking the “View 3D Structure.” In addition, “Comparison/Survival” was selected to assess the prognostic value of somatic mutation frequency and microsatellite instability.

### Immune Cell Infiltration Analysis

The associations between the ADAM12 expression level and the abundances and corresponding specific markers of immune cells were investigated using the TIMER2.0 and GEPIA databases. Next, TISIDB (http://cis.hku.hk/TISID B/index.php), a web portal for tumor and immune system interactions (26), was applied to determine the correlation between the ADAM12 expression level and immune components, including lymphocytes, immunomodulators, and chemokines.

### ADAM12-Related Gene Enrichment Analysis

STRING (http://string-db.org) was applied to construct the protein–protein interaction network of ADAM12 and explore the interacting proteins (27). The minimum required interaction score was set to a medium confidence of 0.400, and the maximum number of interactors was ≤50. The 50 interactors that were most strongly associated with ADAM12 were collected and set as target ADAM12-binding proteins. Second, the ADAM12 gene was entered into the “Query Search” module of GEPIA, and the top 100 ADAM12-related target genes were collected (genes correlated with ADAM12 expression).

Metascape (https://metascape.org), an effective tool for experimental biologists to analyze big data (28), was applied to further analyze the functional enrichment of related genes. Then, an interactive Venn diagram was created to compare the genes between target ADAM12-binding proteins and genes correlated with ADAM12 expression using Bioinformatics, an online drawing software package (http://www.bioinformatics.com.cn/). Pearson’s correlation analysis was conducted between ADAM12 and the correlated genes using the “Gene_Corr” module of TIMER2.0, and an expression heatmap was created to assess the expression patterns of each gene in different cancer types using bioinformatics. In addition, we evaluated the effect of the expression of the correlated genes on the OS/disease-free survival (DFS) of patients with CRC using GEPIA. If the P value was less than 0.01, it was considered significant.

## Results

### Overexpression of ADAM12 in COAD

**Figure 1:**
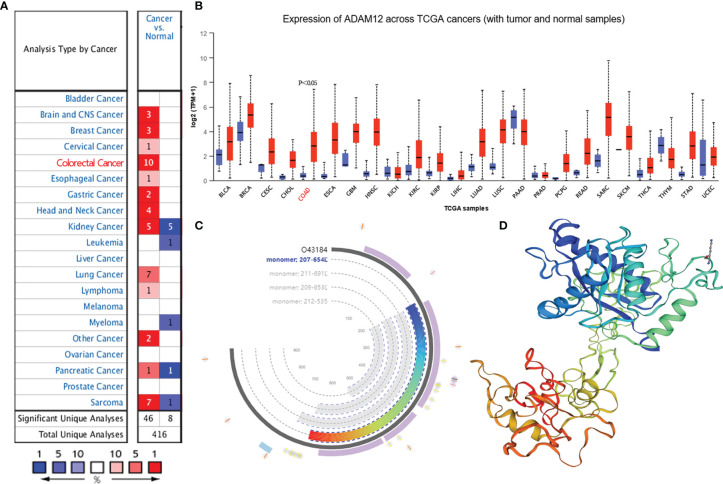
ADAM12 expression in multiple cancers and its protein model and 3D structure. **(A, B)** ADAM12 mRNA expression levels in different cancer samples were explored using the Oncomine and UALCAN databases. **(C)** The ADAM12 protein model was analyzed using the SWISS MODEL. **(D)** The 3D structure of ADAM12 was analyzed using the SWISS MODEL.

Differences in ADAM12 expression between tumor and normal tissues from patients with different cancers were analyzed using the Oncomine database. ADAM12 mRNA was overexpressed in tumor tissues, such as breast cancer, cervical cancer, CRC, and esophageal cancer. However, its expression in leukemia and myeloma was low (**Figure 1A**). UALCAN was used to further analyze ADAM12 expression in human cancers. As a result, an upregulation of BLCA, BRCA, CESC, CHOL, COAD, ESCA, GBM, HNSC, KIRC, LUAD, LUSC, PCPG, PCPG, READ, SARC, SKCM, and STAD expression and a reduced expression of PAAD, THYM, and UCEC were observed. Nevertheless, no significant differences were observed between tumor and normal tissues from patients with KICH, LIHC, PRAD, or THCA (**Figure 1B**). The ADAM12 protein model and 3D structure are shown in **Figures 1C, D**, respectively. The abbreviations for each cancer are summarized in **Supplementary Material 1 (S1)**.

**Figure 2:**
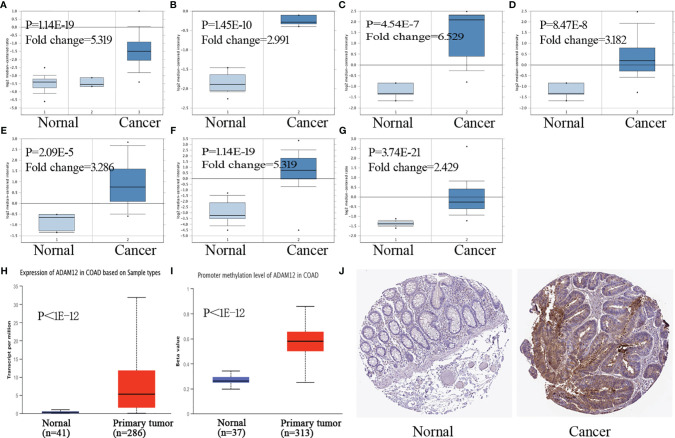
ADAM12 expression in COAD tissues. **(A–G)** ADAM12 expression levels in COAD samples were investigated using the Oncomine database. **(H, I)** Expression and promoter methylation levels of ADAM12 in COAD were analyzed based on tissue samples present in the UALCAN database. **(J)** Images of immunohistochemical staining showing ADAM12 protein expression in colorectal tissue from The Human Protein Atlas.

Moreover, we investigated ADAM12 expression levels in COAD using Oncomine. Compared with the corresponding normal tissue samples, ADAM12 overexpression was observed in colon cancer tissues, including COAD, colon mucinous adenocarcinoma, rectal adenocarcinoma, and cecum adenocarcinoma (**Figures 2A–G**). The promoter methylation level in COAD was also explored in the TCGA analysis module of the UALCAN database. Compared with the normal tissues, the expression and methylation level of ADAM12 in colon adenocarcinoma were significantly upregulated (**Figures 2H, I**). Images of immunohistochemical staining showing ADAM12 protein expression in colorectal tissues are shown in **Figure 2J**.

### Effect of Clinical Indicators on ADAM12 Expression in Patients With COAD

**Figure 3:**
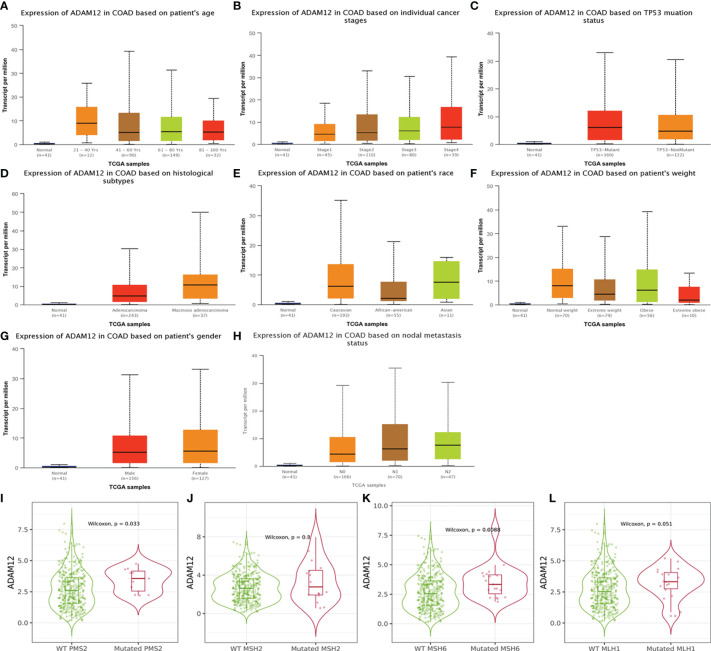
Relationship between ADAM12 expression and clinical indicators in patients with COAD. **(A)** Subgroup analysis of ADAM12 expression in COAD based on patient age. **(B)** Subgroup analysis of ADAM12 expression in COAD based on individual cancer stages. **(C)** Subgroup analysis of ADAM12 expression in COAD based on the TP53 mutation status. **(D)** Subgroup analysis of ADAM12 expression in COAD based on histological subtypes. **(E)** Subgroup analysis of ADAM12 expression in COAD based on patient race. **(F)** Subgroup analysis of ADAM12 expression in COAD based on patient weight. **(G)** Subgroup analysis of ADAM12 expression in COAD based on patient sex. **(H)** Subgroup analysis of ADAM12 expression in COAD based on nodal metastasis status. **(I–L)** Different ADAM12 expression levels in COAD based on PMS2, MSH2, MSH6, and MLH1, respectively.

In our study, we analyzed the effect of different clinical characteristics on ADAM12 expression using the UALCAN database. In terms of age, the statistical analysis revealed significantly lower ADAM12 expression in the healthy group than in patients with COAD in the 21–40, 41–60, 61–80, and 81–100 years age groups (**Figure 3A**). Regarding the individual cancer stages, significant differences were observed between the healthy group and the stage 1–4 group, and the stage 1 group presented with a lower expression than the stage 2–3 group (**Figure 3B**). Patients with CRC carrying a TP53 mutation tended to express ADAM12 at a higher level (**Figure 3C**). In addition, mucinous adenocarcinoma showed higher ADAM12 expression than adenocarcinoma (**Figure 3D**). ADAM12 expression appeared to be higher in Caucasians than in Asians (**Figure 3E**). Different body weights and sexes did not affect ADAM12 expression in patients with CRC (**Figures 3F, G**), while different nodal metastasis statuses affected ADAM12 expression (**Figure 3H**). Differences in ADAM12 expression based on different clinical indicators are summarized in **Table 1**. In addition, high ADAM12 expression was often associated with mutant PMS2 (P=0.033) and MSH6 (P=0.0088) but was unrelated to the mutation status of MLH1 (P=0.3) or MSH2 (P=0.051) (**Figures 3I–L**).

**Table 1:** ADAM12 expression based on different clinical indicators.

Clinical indicators	Number of patients	Comparison	P value
Sample types	41/286 (Normal/Primary tumor)	Normal-*vs*-Primary	***<1E-12***
Individual cancer stages	45/110/80/39 (Stage1/Stage2/Stage3/Stage4)	Stage1-*vs*-Stage2	***3.866500E-02***
		Stage1-*vs*-Stage3	***1.155920E-02***
		Stage1-*vs*-Stage4	1.280250E-01
		Stage2-*vs*-Stage3	7.502400E-01
		Stage2-*vs*-Stage4	9.093400E-01
		Stage3-*vs*-Stage4	9.004800E-01
Patient’s race	193/55/11 (Caucasian/African-American/Asian)	Caucasian-*vs*-African-American	8.786200E-01
		Caucasian-*vs*-Asian	***2.384100E-02***
		African-American-*vs*-Asian	3.204200E-01
Patient’s gender	156/127 (Male/Female)	Male-*vs*-Female	8.391000E-01
Patient’s weight	70/74/56/10 (Normal_weight/Extereme_weight/Obese/Extreme_Obese)	Normal_Weight-*vs*-Extreme_Weight	9.445000E-01
		Normal_Weight-*vs*-Obese	3.961800E-01
		Normal_Weight-*vs*-Extreme_Obese	5.418000E-01
		Normal_Weight-*vs*-Obese	3.961800E-01
		Normal_Weight-*vs*-Extreme_Obese	5.418000E-01
		Extreme_Weight-*vs*-Obese	5.236400E-01
		Extreme_Weight-*vs*-Extreme_Obese	6.754600E-01
Patient’s age	12/90/149/32 (21-40 Yrs/41-60 Yrs/61-80 Yrs/81-100 Yrs)	Age(21-40Yrs)-*vs*-Age(41-60Yrs)	1.110160E-01
		Age(21-40Yrs)-*vs*-Age(61-80Yrs)	5.757800E-01
		Age(21-40Yrs)-*vs*-Age(81-100Yrs)	2.403000E-01
		Age(41-60Yrs)-*vs*-Age(61-80Yrs)	1.879600E-01
		Age(41-60Yrs)-*vs*-Age(81-100Yrs)	***8.783900E-03***
		Age(61-80Yrs)-*vs*-Age(81-100Yrs)	***3.109600E-02***
Histological subtype	243/37 (Adenocarcinoma/Mucinous-adenocarcinoma)	Adenocarcinoma-*vs*-Mucinous-adenocarcinoma	***2.528300E-02***
Nodal metastasis status	166/70/47 (N0/N1/N2)	N0-*vs*-N1	4.218800E-01
		N0-*vs*-N2	2.171400E-01
		N1-*vs*-N2	4.514800E-01
TP53 mutation status	160/122 (Mutant/Non-Mutant)	Mutant-*vs*-Non-Mutant	8.211200E-01

Normal_weight, BMI greater than equal to 18.5 and BMI less than 25; Extereme_weight, BMI greater than equal to 25 and BMI less than 30; Obese, BMI greater than equal to 30 and BMI less than 40; Extreme_Obese, BMI greater than 40; N0, No regional lymph node metastasis; N1, Metastases in 1 to 3 axillary lymph nodes; N2, Metastases in 4 to 9 axillary lymph nodes; N3, Metastases in 10 or more axillary lymph nodes; TP53 mutation status is obtained from TCGA whole exome sequencing data, and the sample with/without TP53 mutation was matched with RNA-seq data. The bold italics indicate statistical significance.

### Relationship Between ADAM12 Expression and Prognosis in Patients With COAD

**Figure 4:**
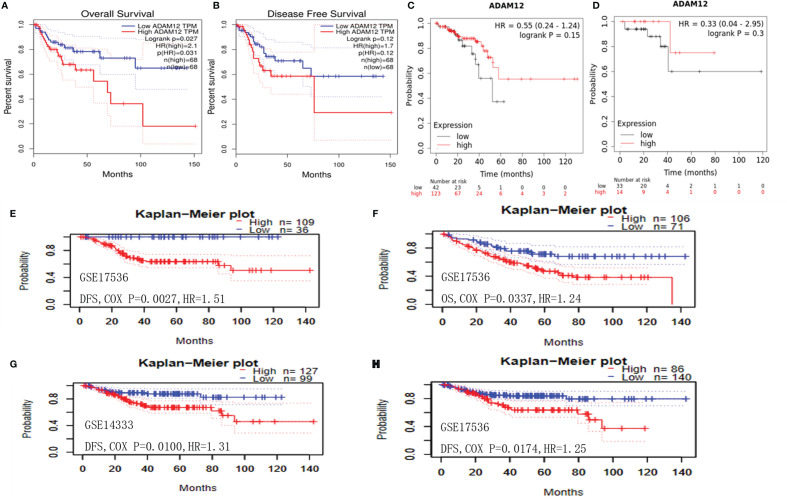
Relationship between ADAM12 expression and prognostic value in patients with COAD. **(A)** OS and **(B)** DFS of patients with COAD based on ADAM12 expression determined using GEPIA. **(C)** OS and **(D)** DFS of patients with COAD based on ADAM12 expression determined using the Kaplan–Meier Plotter. **(E, G, H)** DFS and **(F)** OS of patients with COAD based on ADAM12 expression determined using the PrognoScan database. OS, overall survival; DFS, disease-free survival.

The correlation between ADAM12 expression and prognosis in patients with CRC was subsequently investigated using different databases. Using the GEPIA method, low ADAM12 expression significantly correlated with a longer OS (n = 136, HR = 2.1, p = 0.027) (**Figure 4A**); in contrast, a significant difference was not observed for DFS (n = 136, HR = 1.7, p = 0.12) (**Figure 4B**). Interestingly, ADAM12 was not significantly associated with prolonged OS (n = 165, HR = 0.55, p = 0.15) or DFS (n = 47, HR = 0.33, p = 0.3) (**Figures 4C, D**) as determined using the Kaplan–Meier Plotter.

The PrognoScan database was applied to further evaluate the relationships between ADAM12 expression and prognosis. Unexpectedly, we newly identified some studies (GSE17536-Smith and GSE14333-Jorissen) that showed a correlation between low ADAM12 expression and a shorter OS (n = 177, HR = 0.034, COX-P = 0.042) and DFS (n = 145, HR = 0.41, COX-P = 0.0027; n = 226, HR = 0.27, COX-P = 0.010; n = 226, HR = 0.23, COX-P = 0.017) for patients with COAD (**Figures 4E–H**). In conclusion, based on the aforementioned databases, we discussed the effect of ADAM12 expression on patient prognosis and survival and proposed that ADAM12 is a factor associated with an adverse prognosis in patients with CRC, suggesting that it is a potential target worthy of further study.

### Analysis of Alterations in the ADAM12 Gene

**Figure 5:**
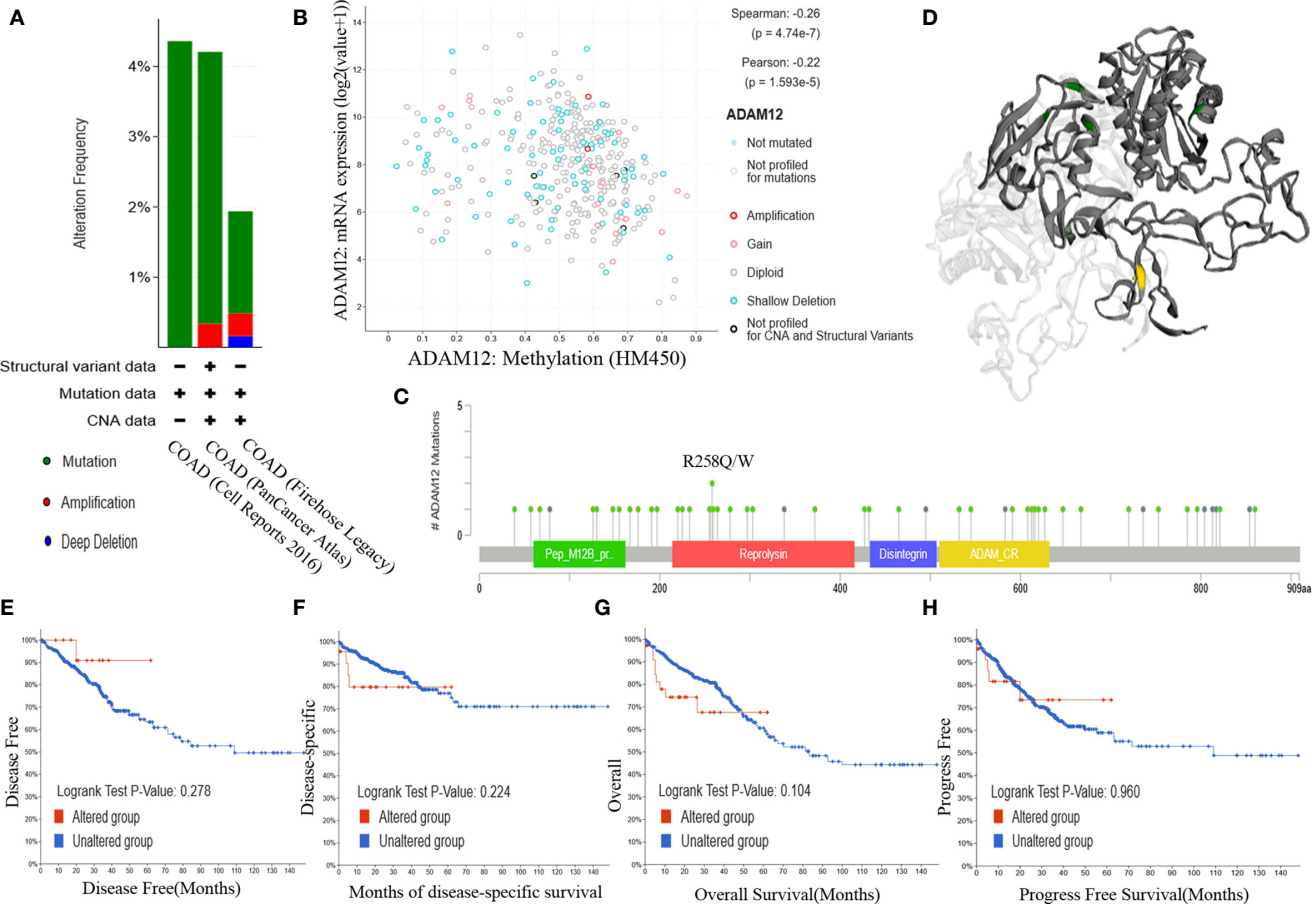
Analysis of ADAM12 genetic alterations using the cBioPortal database. **(A)** Alteration frequency of ADAM12 in different COAD studies. **(B)** Methylation level of ADAM12 based on 372 COAD samples. **(C)** Mutation diagram providing information on the mutation sites, mutation types, and the number of cases, and the results were colored with respect to the corresponding mutation types. **(D)** 3D structure of the ADAM12 protein at the R258Q/W site. Relationships between the mutation status and **(E)** OS, **(F)** DFS, **(G)** progression-free survival, and **(H)** disease-specific survival for patients with colorectal cancer. OS, overall survival; DFS, disease-free survival.

Genetic alteration is proven to be an essential factor affecting the occurrence and progression of different cancers. In our paper, we investigated the relationship between the genetic alteration status and ADAM12 expression in patients with CRC in TCGA cohorts. As a result, approximately 3.2% of COAD samples had somatic mutations in ADAM12. Mutation type copy number variation was the primary type identified in CRC. Amplification and deep deletion types were detected, and the proportions of these mutations are shown in **Figures 5A, B**. Information on the mutation sites, mutation types, and the number of cases is shown in the mutation diagram, which is colored with respect to the corresponding mutation types (**Figure 5C**). In patients with CRC, 61 mutations in the ADAM12 gene were identified, including 8 duplicate mutations in patients with multiple samples. Moreover, we intuitively identified that the R258Q/W alteration was in the reprolysin domain, and the 3D structure of the ADAM12 protein at the R258Q/W site is shown in **Figure 5D**. Additionally, we further evaluated whether alterations in the ADAM12 gene in CRC were associated with patient survival. However, our results indicated that more genetic alterations were not linked to a poor OS (P = 0.221), DFS (P = 0.355), progression-free survival (P = 0.924), or disease-specific survival (P = 0.231) for patients with CRC (**Figures 5E–H**).

### Related Gene Enrichment Analysis of ADAM12

**Figure 6:**
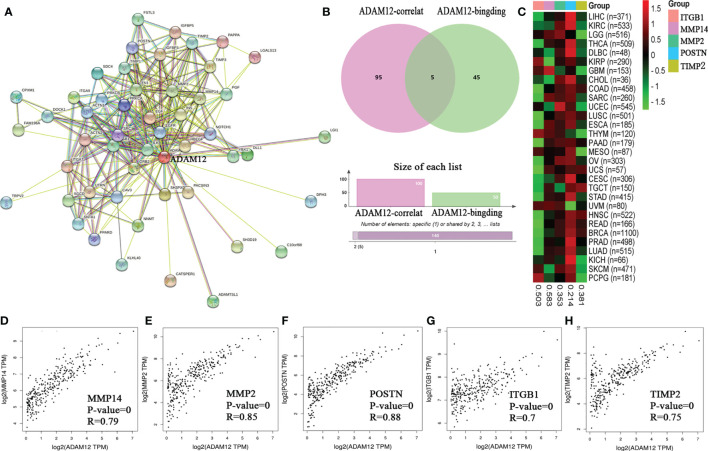
The protein–protein interaction network of ADAM12 was analyzed using the STRING tool. **(A)** Protein–protein interaction network based on ADAM12-binding proteins and genes correlated with ADAM12 expression. **(B)** Interactive Venn diagram to analyze the difference between ADAM12-binding proteins and genes correlated with ADAM12 expression. **(C)** The corresponding heatmap data from the pancancer dataset. Correlations between the expression of ADAM12 and **(D)** MMP14, **(E)** MMP2, **(F)** POSTN, **(G)** ITGB1, and **(H)** TIMP2.

Target ADAM12-binding proteins and genes correlated with ADAM12 expression were identified using the STRING and GEPIA databases to functionally analyze the potential mechanism of ADAM12 in tumor progression. Fifty ADAM12-binding proteins and the top 100 genes correlated with ADAM12 expression were searched. All genes that were screened are summarized in **Supplementary Material 2 (S2)**, and the interaction network of ADAM12-binding proteins and genes correlated with ADAM12 expression is shown in **Figure 6A**. Using the interactive Venn diagram to analyze the difference between the two datasets, our results indicated that ADAM12 expression in COAD was strongly correlated with the Periostin (POSTN), Matrix metallopeptidase 14 (MMP14), Integrin subunit beta 1 (ITGB1), TIMP metallopeptidase inhibitor 2 (TIMP2), and Matrix metallopeptidase 2 (MMP2) genes (**Figure 6B**). According to the Human Protein Atlas database, the main functions of these interacting genes are tissue development and regeneration, embryo development, tissue repair, and angiogenesis, among others. In addition, the expression heatmap also showed that the ADAM12 gene was positively correlated with the five interacting genes listed above in 30 different cancers (**Figure 6C**).

**Figure 7:**
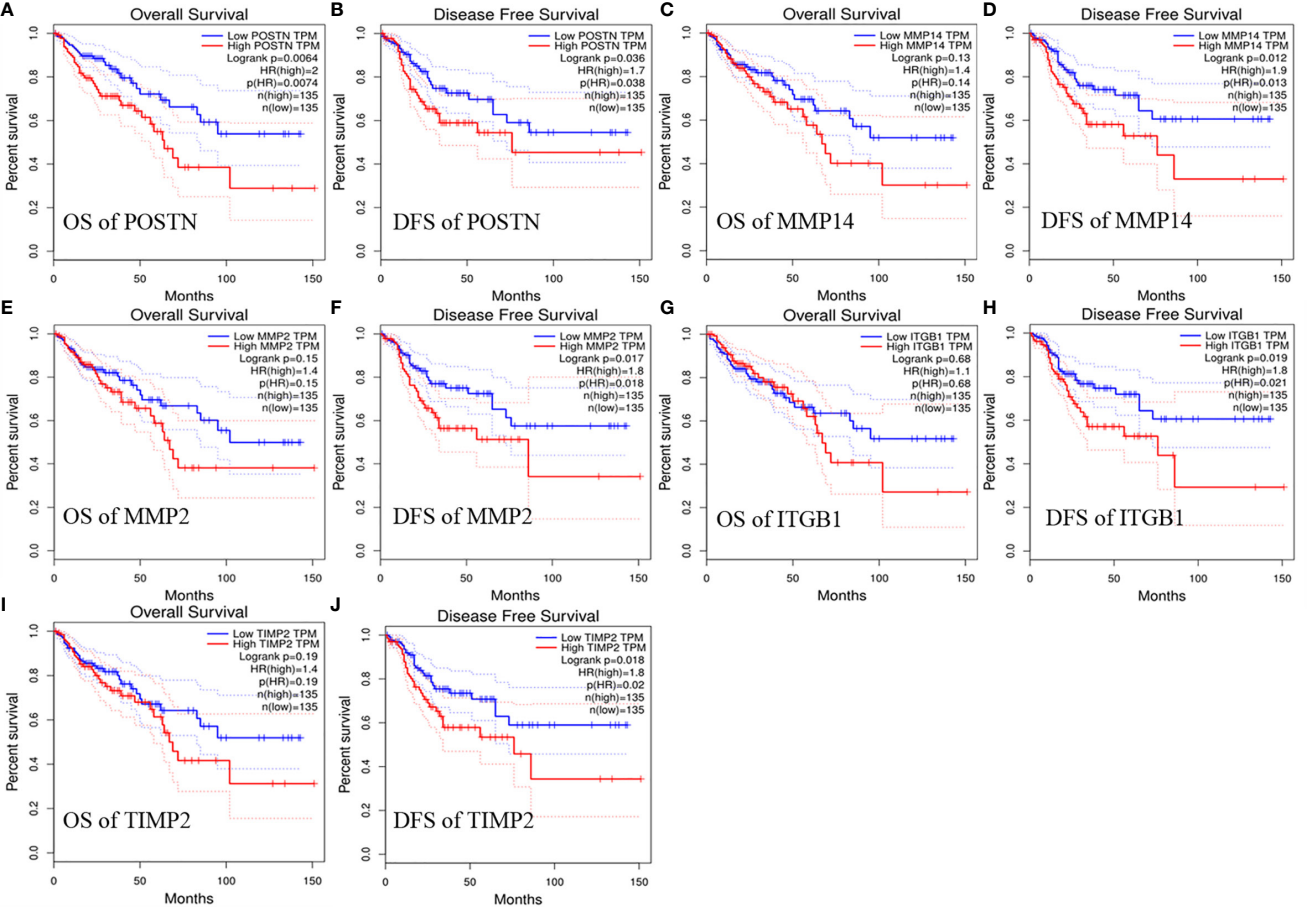
Relationship between selected target gene expression and prognostic value in patients with COAD. **(A)** OS and **(B)** DFS of patients with COAD based on POSTN expression detected using GEPIA. **(C)** OS and **(D)** DFS of patients with COAD based on MMP14 expression detected using GEPIA. **(E)** OS and **(F)** DFS of patients with COAD based on MMP2 expression detected using GEPIA. **(G)** OS and **(H)** DFS of patients with COAD based on ITGB1 expression detected using GEPIA. **(I)** OS and **(J)** DFS of patients with COAD based on TIMP2 expression detected using GEPIA.

We evaluated the relationship between the expression of these interacting genes and the target gene ADAM12 in COAD using the TIMER database and OS and PFS with the GEPIA database to obtain a deeper understanding of the relationships and their effects on prognosis. On the one hand, the analysis results showed that ADAM12 expression was closely associated with the expression levels of POSTN (Cor=0.913, P=3.52e-180), MMP14 (Cor=0.805, P=1.40e-105), MMP2 (Cor=0.876, P=7.5e-147), ITGB1 (Cor=0.572, P=3.32e-41), and TIMP2 (Cor=0.866, P=1.30e-139) (**Figures 6D–H**). On the other hand, high POSTN expression was significantly associated with shorter OS (n=270, HR=2, P=0.0064) and DFS (n=270, HR=1.7, P=0.036). Regarding the MMP14, MMP2, ITGB1, and TIMP2 genes, higher expression of these genes led to shorter DFS (n=270, HR=1.9, P=0.012; n=270, HR=1.8, P=0.017; n=270, HR=1.8, P=0.019; n=270, HR=1.8, P=0.018); in contrast, no significant difference was observed in OS (n=270, HR=1.4, P=0.13; n=270, HR=1.4, P=0.15; n=270, HR=1.1, P=0.28; n=270, HR=1.4, P=0.19) (**Figure 7**).

**Figure 8:**
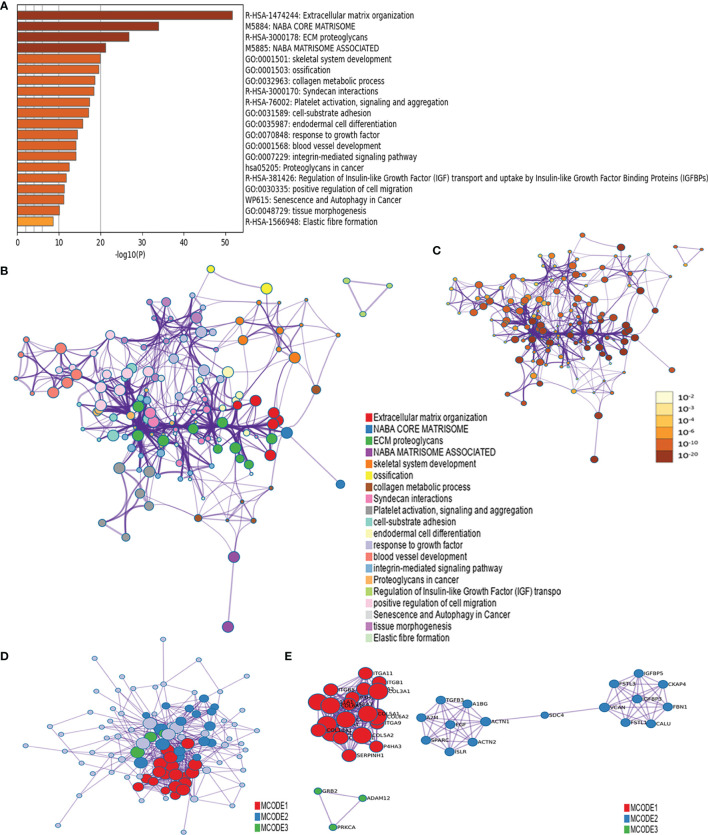
The functional enrichment analysis of ADAM12 was performed with the Metascape tool. **(A)** GO enrichment analysis data indicated that these genes were mainly enriched in extracellular matrix (ECM) organization, NABA CORE MATRISOME, ECM proteoglycans, NANA MATRISOME ASSOCIATED, and skeletal system development. **(B)** The KEGG pathway analysis showed enrichment in extracellular matrix organization, NABA CORE MATRISOME, ECM proteoglycans, NANA MATRISOME ASSOCIATED, and skeletal system development. **(C)** Nodes in the same enrichment network colored by P value, as shown in the legend. The darker the color, the more significant the node (see legend for P value ranges). **(D, E)** The protein–protein interaction network showed enrichment in extracellular matrix organization, NABA CORE MATRISOME, ECM proteoglycans, NANA MATRISOME ASSOCIATED, and skeletal system development.

We combined the ADAM12-binding proteins and the top 100 genes correlated with ADAM12 expression to conduct Gene Ontology (GO) and Kyoto Encyclopedia of Genes and Genomes (KEGG) pathway analyses in Metascape and further predicted the functions and pathways in which ADAM12 was enriched. The GO enrichment analysis data indicated that these related genes were mainly enriched in extracellular matrix (ECM) organization, NABA CORE MATRISOME, ECM proteoglycans, NANA MATRISOME ASSOCIATED, and skeletal system development. Moreover, ossification, collagen metabolic process, syndecan interactions, platelet activation, signaling and aggregation, cell-substrate adhesion, endodermal cell differentiation, response to growth factor, blood vessel development, proteoglycans in cancer, regulation of insulin-like growth factor (IGF) transport and uptake, and other pathways were also involved in the regulation of ADAM12-interacting genes (**Figure 8A**). Furthermore, the results of the protein–protein interaction network and KEGG pathway analysis also showed enrichment in extracellular matrix organization, NABA CORE MATRISOME, ECM proteoglycans, NANA MATRISOME ASSOCIATED, and skeletal system development (**Figures 8B–E**).

### Relationship Between ADAM12 and Immune Cell Infiltration in COAD

**Figure 9:**
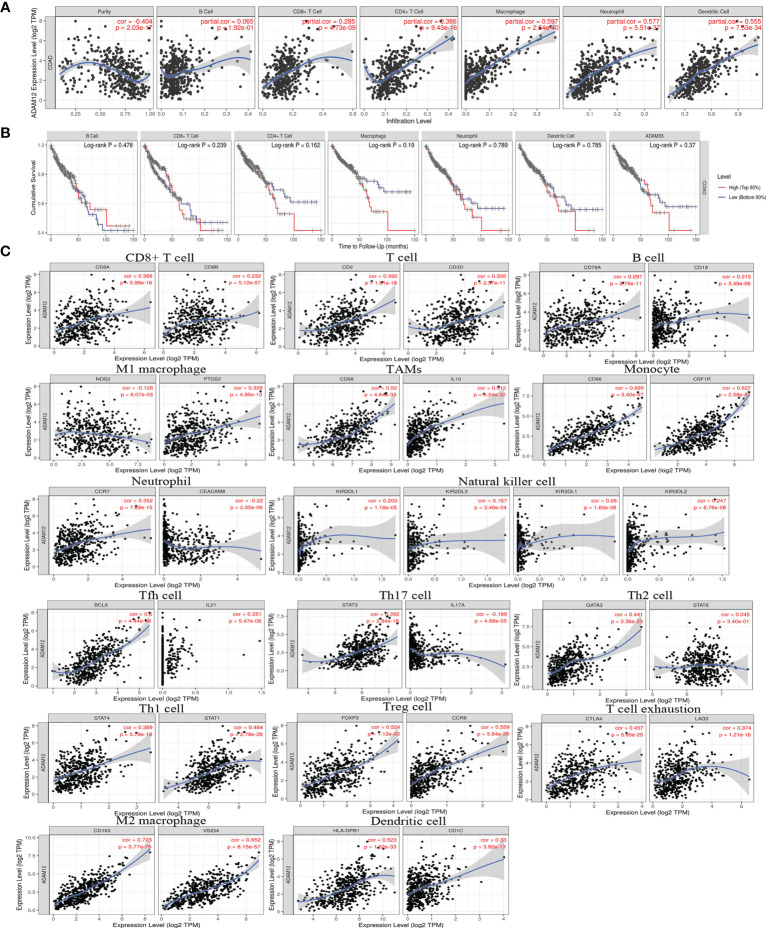
Correlation analysis between the ADAM12 expression level and immune cell infiltration in COAD using the TIMER database. **(A)** Relationship between the ADAM12 expression level and the immune cell infiltration level. **(B)** Relationship between immune cell abundance and survival of patients with COAD. **(C)** Correlations between ADAM12 expression and immune cell-specific marker expression.

The correlation between ADAM12 expression and immune cell infiltration in COAD was analyzed using the TIMER database. In our results, ADAM12 expression was significantly negatively correlated with tumor purity (Cor=-0.404, P=2.03e-17) and was significantly positively correlated with CD8+ T cells (partial cor=0.285, P=4.73e-09), CD4+ T cells (partial cor=0.386, P=9.43e-16), macrophages (partial cor=0.597, P=2.64e-40), neutrophils (partial cor=0.557, P=5.51e-37), and dendritic cells (partial cor=0.555, P=7.53e-34) (**Figure 9A**). However, the correlation between ADAM12 expression and B cells was not significant (partial cor=0.065, P=1.92e-01). Nevertheless, we further generated Kaplan–Meier plots using the TIMER database to explore whether a high level of immune cell infiltration was linked to a longer survival time of patients with COAD. The results indicated no significant associations [B cells (P=0.478), CD8+ T cells (P=0.239), CD4+ T cells (P=0.162), macrophages (P=0.19), neutrophils (P=0.789), dendritic cells (P=0.785), or ADAM12 (P=0.261)] (**Figure 9B**).

Additionally, correlations between ADAM12 expression and the expression of immune cell-specific markers were also investigated using the TIMER and GEPIA databases, including CD8+ T cells (CD8A and CD8B), T cells (CD2 and CD3D), B cells (CD79A and CD19), monocytes (CD86 and CSF1R), tumor-associated macrophages (TAMs) (CD68 and IL10), M1 macrophages (NOS2 and PTGS2), M2 macrophages (CD163 and VSIG4), natural killer cells (KIR2DL1, KIR2DL3, KIR3DL1, and KIR3DL2), dendritic cells (HLA-DPB1 and CD1C), Th2 cells (GATA3 and STAT6), Tfh cells (BCL6 and IL21), Th1 cells (STAT4 and STAT1), Th17 cells (STAT3 and IL17A), neutrophils (CCR7 and CEACAM8), Treg cells (FOXP3, CCR8), and T cell exhaustion (CTLA4 and LAG3). Based on the TIMER database, we observed a significant positive correlation between ADAM12 and most markers, including CD8A, CD8B, CD2, CD3D, CD79A, CD19, CD86, CSF1R, CD68, IL10, PTGS2, CD163, VSIG4, CCR7, KIR2DL1, KIR2DL3, KIR3DL1, KIR3DL2, HLA-DPB1, CD1C, STAT4, STAT1, GATA3, STAT6, BCL6, IL21, STAT3, FOXP3, CCR8, CTLA4, and LAG3, and a significant negative correlation between ADAM12 and the markers NOS2, CEACAM8, and IL17A. All results obtained from the TIMER database are shown **Figure 9C**. In addition, we obtained similar results using the GEPIA database, and our analysis results are shown in **Table 2**. Therefore, these results strongly suggested that the ADAM12 gene mediated the regulation of immune cell infiltration in CRC, thereby promoting immune escape and the chemotherapy resistance of tumor cells.

**Table 2:** Correlation between ADAM12 and immune cell-specific markers in COAD.

Immune cell types	Markers	R	P value
CD8+ T cell	CD8A	0.15	***0.0071***
	CD8B	0.02	0.72
T cell	CD2	0.27	***9E-07***
	CD3D	0.17	***0.0028***
B cell	CD79A	0.049	0.38
	CD19	0.056	0.32
Monocyte	CD86	0.62	***0.00***
	CSF1R	0.62	***0.00***
TAM	CD68	0.47	***0.00***
	IL10	0.52	***0.00***
M1 macrophage	NOS2	-0.08	***0.15***
	PTGS2	0.32	***1E-08***
M2 macrophage	CD163	0.75	***0.00***
	VSIG4	0.51	***0.00***
Neutrophil	CCR7	0.17	***0.0028***
	CEACAM8	-0.026	0.64
Natural killer cell	KIR2DL1	0.31	***2.7E-08***
	KIR2DL3	0.24	***1.5E-05***
	KIR3DL1	0.066	0.24
	KIR3DL2	0.32	***4E-09***
Dendritic cell	HLA-DPB1	0.38	***1.4E-12***
	CD1C	0.19	***0.00055***
Th1 cell	STAT4	0.34	***3.5E-10***
	STAT1	0.25	***5.9E-06***
Th2 cell	GATA3	0.5	***0.00***
	STAT6	0.0049	0.93
Tfh cell	BCL6	0.68	***0.00***
	IL21	0.17	***0.0032***
Th17 cell	STAT3	0.36	***4.7E-11***
	IL17A	-0.079	0.16
Treg cell	FOXP3	0.44	***2.2E-16***
	CCR8	0.49	***0.00***
T cell exhaustion	CTLA4	0.34	***3.2E-10***
	LAG3	0.088	0.12

TAM, tumor-associated macrophage. The bold italics indicate statistical significance.

### Relationship Between ADAM12 Expression and Immune Molecules

We explored the relationships between the ADAM12 expression level and immune components, such as lymphocytes, immunomodulators, and chemokines, in patients with COAD using the TISIDB tool to obtain a deeper understanding of the association of ADAM12 with immune cell infiltration.

**Figure 10:**
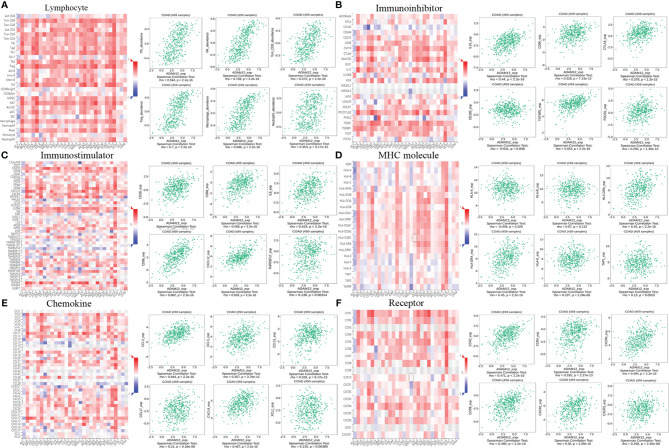
Relationship between ADAM12 expression and immune components in patients with COAD, including lymphocytes, immunomodulators, and chemokines. **(A)** Relationship between the ADAM12 expression level and lymphocytes. **(B–D)** Relationship between the ADAM12 expression level and immunomodulators. **(E, F)** Correlations between ADAM12 expression levels and chemokines.

First, the relationships between the abundance of tumor-infiltrating lymphocytes and ADAM12 expression levels were investigated to examine which types of TILs might be regulated by the ADAM12 gene. The outcomes indicated that the ADAM12 expression level was positively correlated with Tfh cells (rho=0.594, P<2.2e-16), Tcm_CD8 cells (rho=0.572, P<2.2e-16), Treg cells (rho=0.7, P<2.2e-16), NK cells (rho=0.728, P<2.2e-16), and MDSCs (rho=0.608, P<2.2e-16) (**Figure 10A**). However, the abundance of monocytes was not regulated by the ADAM12 gene (rho=-0.002, P=0.96) (**Figure 10A**).

Next, we evaluated the relations between three different types of immunomodulators and ADAM12 expression, and these immunomodulators included immunoinhibitors, immunostimulators, and major histocompatibility complex molecules. The immunomodulators were collected from the study by Charoentong. Correlations between immunoinhibitors, including IL10_exp, CD96_exp, CTLA4_exp, CD160_exp, TGFBR1_exp, and PDCD1_exp, and ADAM12 are shown in **Figure 10B**. Correlations between immunostimulators, including CD28_exp, CD80_exp, CD86_exp, CXCL12_exp, IL6_exp, and TNFRSF17_exp, and ADAM12 are shown in **Figure 10C**. Correlations between major histocompatibility complex molecules, including HLA-A_exp, HLA-B_exp, HLA-DOA_exp, HLA-DRA_exp, HLA-E_exp, and TAP1_exp, with ADAM12 are shown in **Figure 10D**.

Finally, we explored the relationships between chemokines, receptors, and ADAM12 expression. Correlations between chemokines, including CCL3_exp, CCL5_exp, CCCL11_exp, CCL17_exp, CXCL9_exp, and XCL1_exp, and ADAM12 are shown in **Figure 10E**. Correlations between receptors, including CCR2_exp, CCR4_exp, CCR8_exp, CXCR1_exp, CXCR4_exp, and CXCR5_exp, and ADAM12 are shown in **Figure 10F**.

The results strongly suggested that ADAM12 regulated various immune molecules in the tumor microenvironment of CRC through many pathways, thereby affecting immune cell infiltration.

## Discussion

COAD is the most common histological type of CRC. In the current clinical treatment context, radical resection is the most conventional treatment for CRC. However, surgical treatment is often limited by the location of the tumor and the depth of invasion, and the complete removal of the cancer cells during tumor resection appears impossible, which often leads to postoperative tumor recurrence or metastasis. Recently, with the rapid development of molecular biology and tumor immunology, a series of therapeutic methods, including targeted therapy, chemotherapy, radiotherapy, and immunotherapy, have become new therapeutic options for many types of cancer, effectively improving the survival of patients with advanced-stage tumors (29).

Notably, immunotherapy has become a hot spot in drug application and research and is a promising treatment choice (30). Unfortunately, immunotherapy only produces a good response in a small subset of CRCs that exhibit microsatellite instability (MSI), but most CRCs are microsatellite stable (MSS). Studies have shown that the differences between MSI and MSS tumors in response to immune checkpoint inhibitors are mainly attributed to the differences in the immune microenvironment of primary tumors. Compared with MSS tumors, MSI tumors have higher levels of immune stimulators and chemokines, including IFN-GAMMA, IL-15, CCL3, CXCL16, and CXCL14 (31). In the tumor microenvironment, more bioactive immune cells increase major histocompatibility complex 1 and immune checkpoint-related protein expression in tumor cells and simultaneously inhibit tumor immune escape by secreting a variety of immunosuppressive cytokines and receptors to overcome the resistance of CRC tumor MSS subtypes to immune checkpoint inhibitors. In the future, methods to induce the activation of the immune microenvironment, screen groups who might benefit, and select the best combination therapy will become a great challenge for clinicians and researchers (32, 33).

In recent years, studies aiming to mine reliable immune-related genes in tumors, screen the dominant population, and predict the efficacy of immune checkpoint inhibitors have been reported (34–36). In our paper, we reported a potential prognostic biomarker associated with immune cell infiltration in patients with COAD.

First, we analyzed ADAM12 expression and the association of its aberrant expression with patient prognosis. Compared with normal samples, ADAM12 was expressed at high levels in most tumors, such as BLCA, BRCA, CESC, CHOL, and COAD. In addition, ADAM12 overexpression was also observed in CRC tissues, and higher expression was linked to shorter OS or PFS. Additionally, ADAM12 expression was affected by patient age, individual cancer stages, TP53 mutation status, and other clinical factors. A literature search indicated that our findings were supported by some studies (37–40), whereas its role in the development of cancer remains an area of limited research. According to our research results, the ADAM12 protein is upregulated in CRC and leads to a poorer prognosis, which suggests a potential key role for ADAM12 and prompts us to investigate its biological function in COAD. ADAM12 seems to be a highly interesting research topic not only in terms of epigenetic changes but also with regard to the role of ADAM12 in the pathogenesis and progression of cancer and in the relationship between immune cell infiltration.

According to previous studies, matrix metalloproteinases are mainly involved in the degradation of ECM and the regulation and processing of growth factors, chemokines, and cytokines, thus playing an important role in tumor progression. Both ADAM12 and matrix metalloproteinase proteins are members of the protease family and promote tumor cell invasion by regulating cell–cell or cell–extracellular matrix interactions. ADAM12 and MMP14 are significantly correlated with cavernous sinus invasion in patients with pituitary adenomas (41). Furthermore, ADAM12 reduces tumor cell apoptosis and promotes tumor growth by redistributing and activating MMP14, resulting in gelatin degradation (42). In the present study, the results shown in the interactive Venn diagram revealed that ADAM12 expression was strongly correlated with MMP14, MMP2, and TIMP2 expression. Therefore, we hypothesized that ADAM12 may function as an oncogene in most tumors, especially in CRC.

Then, the GO enrichment analysis indicated that interacting genes correlated with extracellular matrix organization, ECM proteoglycans, signaling and aggregation, cell-substrate adhesion, endodermal cell differentiation, response to growth factor, blood vessel development, proteoglycans in cancer, regulation of insulin-like growth factor transport, and uptake. The research findings from several related studies are consistent with the results of our GO analysis.

The tumor microenvironment, which is composed of extracellular matrix, soluble factors (growth factors/cytokines/cytokines), and stromal cells, regulates the growth and proliferation of tumor cells and is certainly considered a possible mechanism of tumor progression. Luo et al. (43) indicated that ADAM12 was overexpressed in esophageal cancer. In addition, effective activation of the positive feedback loop composed of ADAM12 and focal adhesion kinase enhances the interaction between tumor cells and the extracellular matrix, thus promoting the metastasis of esophageal cancer. Certainly, the mRNA expression of TWIST1 and ADAM12 is strongly correlated in human breast tumors, and the TWIST1 transcription factor promotes tumor invasion and metastasis by inducing epithelial-mesenchymal transformation and invadopodia-mediated degradation of the extracellular matrix (44). Therefore, ADAM12 is considered correlated with the extracellular matrix and plays a role in tumor progression in the tumor microenvironment; however, these findings require further study for confirmation.

Mutations in the components of the EGFR pathway are known to be associated with human cancers. In the present study, we observed a robust relationship between aberrant ADAM12 expression and the EGFR pathway in patients with COAD. By conducting a literature search, we retrieved studies on the interaction between ADAM12 and the EGFR pathway. In a study on pituitary adenoma, Wang et al. (10) showed that ADAM12 induces EMT through the EGFR/ERK signaling pathway and promotes the migration, invasion, and proliferation of pituitary adenoma cells *in vivo* and *in vitro*, thus suggesting that ADAM12 potentially represents a valuable therapeutic target for pituitary adenoma. Other studies have shown that ADAM12 is associated with the EGFR signaling pathway and mediates tumor progression through interactions with other factors (45–48). Unfortunately, few reports are available on the interaction between ADAM12 and the EGFR pathway in CRC.

In addition to the EGFR signaling pathway, ADAM12 also induces the formation of new blood vessels in tumors, consistent with the results of our analysis. Recently, ADAM12 was reported to play an important role in tumor neovascularization and/or tumor cell extravasation by activating the shedding of several membrane-anchored proteins, such as sonic hedgehog, Delta-like 1, and certain epidermal growth factor receptor ligands (49). We hypothesized that ADAM12 affects some cytokines required for angiogenesis in the tumor microenvironment *via* specific signaling pathways, such as vascular cell adhesion molecule 1 and vascular endothelial cadherin, and mediates tumor angiogenesis to subsequently promote tumor progression (48). Moreover, activation of pathways associated with insulin resistance and insulin-like growth factor (IGF) appears to be associated with metabolic syndrome and CRC development, which is an interesting result. ADAM12 and ADAM28 regulate the bioavailability of growth factors through the IGF signaling pathway and affect the canceration process of CRC (50), consistent with the results of our GO analysis. Therefore, our results clearly reveal that ADAM12 is involved in many aspects of cancer development.

Another major finding from our research was that ADAM12 expression was related to the infiltration of diverse immune cell types in COAD. Our results based on data mining indicated that ADAM12 expression was significantly positively correlated with CD8+ T cells, CD4+ T cells, macrophages, neutrophils, and dendritic cells. However, the correlation between ADAM12 expression and B cells was not significant, suggesting that the tumor was heterogeneous in the antigens presented to cells recruited to the TME. As expected, the correlations between ADAM12 expression and the expression of specific markers, such as CD8A, CD68, CD163, NOS2, and IL10, were successfully observed using the TIMER and GEPIA databases. Nevertheless, further analysis in the present study showed no significant relationship between the immune cell abundance and survival of patients with CRC. Taken together, these findings suggested that ADAM12 played an important role in the recruitment and regulation of infiltrating immune cells in CRC.

Based on the findings described above, the biological function of ADAM12 has been revealed, suggesting that it interacts with the immune microenvironment during CRC progression. An increased CD8+ T lymphocyte density in tumor tissues has been reported to be associated with a reduced risk of recurrence, independent of confounding factors, including DNA mismatch repair defects, POLE mutations, and chromosomal instability (51). Activated CD8+ T cells recruit macrophages by inducing tumor cells to express certain antigen molecules, which is conducive to the immune escape of tumor cells (52). Effective tumor eradication requires tumor-specific CD8+ and CD4+ T cells. We hypothesized that the infiltration of large numbers of CD8+ T lymphocytes may promote the release of certain signals from the cell surface to recruit other upregulated immune cells, including CD4+ T cells, macrophages, neutrophils, and dendritic cells, thereby modulating the interaction between tumor cells and immune cells to regulate the immunotherapy response, which explained the simultaneous aggregation of several immune cells in our results. Moreover, the analysis of results from the TISIDB database showed the associations between the ADAM12 expression level and lymphocytes, immunomodulators, and chemokines in patients with COAD. Overall, our results clearly indicated that ADAM12 is closely related to the immune cell penetration of CRC and may be a new target worthy of further investigation in the field of colorectal tumor immunotherapy.

In summary, our results revealed the abnormal expression of ADAM12 in CRC tissues, and we evaluated the association between high ADAM12 expression and poorer prognosis using an online database. In addition, we evaluated the association between ADAM12 expression levels and immune cell infiltration and immune cell-specific markers and revealed that ADAM12 expression levels were significantly associated with the abundances of various lymphocytes, immunomodulators, and chemokines in COAD tissues. These results strongly suggest that we should detect ADAM12 in postoperative specimens from patients with CRC to better predict the survival time of patients or even to evaluate the status of the immune microenvironment to guide the use of immunotherapy drugs.

Our study still has some limitations. This study was based on the analysis of multiple online databases; the available data were very limited, and some differences in statistical analysis methods were noted among different databases. In addition, we lacked sufficient data to explore the role of ADAM12 in regulating the recruitment of immune cells, and more sophisticated experimental designs are needed to verify these promising results. However, we propose that ADAM12 is an important immune-related factor and a potentially useful and reliable biomarker for guiding immunotherapy and determining the prognosis of patients with CRC after considering the limitations.

## Conclusions

In colorectal tumors, ADAM12 may play vital roles in regulating the ECM and the recruitment of immune cells, and we postulate that it may become a reliable biomarker for the immunotherapy response and prognosis of patients with COAD.

## Data Availability Statement

The datasets presented in this study can be found in online repositories. The names of the repository/repositories and accession number(s) can be found in the article/**Supplementary Material**.

## Author Contributions

Each author made substantial contributions to the conception or design of the work. Study concept and design: ZH and HQ. Acquisition of data and manuscript writing: ZH. Analysis and interpretation: JL, HL, JC, BL, LM, and WW. Study supervision: XM and HQ. All authors contributed to the article and approved the submitted version.

## Funding

This work was supported by the Guangxi Health Department Project (Z2016506); 2019 Guangxi University High-level Innovation Team and the Project of Outstanding Scholars Program, and Guangxi Science and Technology Project (2019AC03004); and Guangxi Science and Technology Base and Talent Project (AD19245197).

## Conflict of Interest

The authors declare that the research was conducted in the absence of any commercial or financial relationships that could be construed as a potential conflict of interest.

## Publisher’s Note

All claims expressed in this article are solely those of the authors and do not necessarily represent those of their affiliated organizations, or those of the publisher, the editors and the reviewers. Any product that may be evaluated in this article, or claim that may be made by its manufacturer, is not guaranteed or endorsed by the publisher.

## Abbreviations

ADAM12, a disintegrin and metalloprotease 12; COAD, colon adenocarcinoma; GEPIA, Expression Profiling Interactive Analysis; ECM, extracellular matrix; CRC, colorectal cancer; OS, overall survival; DFS, disease-free survival; EMT, epithelial mesenchymal transformation; GO, Gene Ontology; KEGG, Kyoto Encyclopedia of Genes and Genomes.
