# Supplementary material for: Upregulation of ADAM12 Is Associated With a Poor Survival and Immune Cell Infiltration in Colon Adenocarcinoma
Source: Front Oncol. 2021 Sep 16;11:729230. doi: 10.3389/fonc.2021.729230 (PMC8483634; doi:10.3389/fonc.2021.729230)
Supplement: Supplementary file 1 [file Table_1.docx]

| S1. The abbreviations for 24 cancers. | |
| --- | --- |
| Abbreviations | Full name |
| BLCA | Bladder urothelial carcinoma |
| BRCA | Breast invasive carcinoma |
| CESC | Cervical squamous cell carcinoma |
| CHOL | Cholangiocarcinoma |
| COAD | Colon adenocarcinoma |
| ESCA | Esophageal carcinoma |
| GBM | Glioblastoma multiforme tumor |
| HNSC | Head and neck squamous cell carcinoma |
| KICH | Kidney chromophobe |
| KIRC | Kidney renal clear cell carcinoma |
| KIRP | Kidney renal papillary cell carcinoma |
| LIHC | Liver hepatocellular carcinoma |
| LUAD | Lung adenocarcinoma |
| LUSC | Lung squamous cell carcinoma |
| PAAD | Pancreatic adenocarcinoma |
| PRAD | Prostate adenocarcinoma |
| PCPG | Pheochromocytoma and paraganglioma |
| READ | Rectal adenocarcinoma |
| SARC | Sarcoma tumor |
| SKCM | Skin cutaneous melanoma |
| THCA | Thyroid carcinoma |
| THYM | Thymoma |
| STAD | Stomach adenocarcinoma |
| UCEC | Uterine corpus endometrial carcinoma |
