# Supplementary material for: Upregulation of ADAM12 Is Associated With a Poor Survival and Immune Cell Infiltration in Colon Adenocarcinoma
Source: Front Oncol. 2021 Sep 16;11:729230. doi: 10.3389/fonc.2021.729230 (PMC8483634; doi:10.3389/fonc.2021.729230)
Supplement: Supplementary file 2 [file Table_2.docx]

| S2. Top 100 expression-correlated genes and 50 ADAM12-bingding proteins. | |
| --- | --- |
| Descriptions | Genes |
| Top 100 expression-correlated genes | COL5A2, COL3A1, COL1A2, COL1A1, COL5A1, LRRC15, POSTN, COL12A1, MXRA5, ADAMTS12, COL6A3, FAP, CTHRC1, CDH11, SPARC, AC093850.2, ASPN, ZNF469, THBS2, COL11A1, MMP14, GXYLT2, RP11-863P13.3, DAMTS2, ANTXR1, RP11-417E7.2, ITGA11, COL10A1, LUM, GPX8, C1QTNF6, MMP2, SFRP2, WISP1, LOXL1, ITGB5, FBN1, PRRX1, PXDN, LOXL2, KIF26B, AC066694.1, HTRA1, OLFML2B, CD276, BGN, AEBP1, PLXDC2, SERPINH1, SULF1, SH3PXD2B, VCAN, MMP11, SEC24D, CMTM3, ITGBL1, CERCAM, CHSY3, CILP, COL8A2, COL6A1, GLT8D2, FKBP7, FAM26E, MRC2, COL6A2, FAM114A1, TGFB3, P4HA3, CALU, ITGB1, THY1, ENAH, RCN3, RP11-426C22.4, GALNT10, CHSY1, INHBA, SEC23A, ISLR, ITPRIPL2, FSTL1, CKAP4, RP3-495K2.2, P3H1, TPM4, LHFPL2, ARF4, CTD-2171N6.1, PPIC, SRPX2, LRRC17, WISP1-OT1, DIRC1, TIMP2, TRAM2, RP11-334E6.12, UBTD2, PODNL1, SEPT11. |
| 50 ADAM12-bingding proteins | EGF, SH3PXD2A, ITGA9, DLL1, ITGB1, SDC4, PRKCB, PRKCA, SRC, PAPPA, LGALS13, ACTN2, ITGA7, IGFBP5, CATSPER1, IGFBP3, UTRN, PACSIN3, HBEGF, GRB2, INS, TIMP3, SH3D19, MMP14, FAM196A, MMP2, FN1, SGCD, NNMT, LGI1, C10orf88, ADAMTSL1, YBX1, ILK, FSTL3, PGF, TIMP1, KLHL40, TIMP2, DOCK1, CPXM1, SNTA1, TRPV2, DPH3, ACTN1, EGFR, CAV3, OTCH1, PPARD, POSTN. |
